# Supplementary material for: A Serial Mediation Model of Insecure Attachment and Psychological Distress: The Role of Dispositional Shame and Shame-Coping Styles
Source: Int J Environ Res Public Health. 2023 Feb 11;20(4):3193. doi: 10.3390/ijerph20043193 (PMC9966328; doi:10.3390/ijerph20043193)
Supplement: Supplementary file 1 [file ijerph-20-03193-s001.zip › ijerph-2146195-supplementary.pdf]

Supplementary Material

Table S1.

Skewness and kurtosis for all measures used in the study (N = 978)

Variables	Skweness	Kurtosis
ASQ ANX	0.322	0.183
ASQ AVD	0.195	−0.098
ESS	0.745	0.182
CoSS AO	0.669	0.246
CoSS AS	0.345	−0.427
CoSS WD	0.466	−0.253
CoSS AV	0.143	0.011
K10	0.688	−0.027

Note. ASQ = Attachment Style Questionnaire; ANX = attachment anxiety; AVD = attachment avoidance; ESS = Experience of Shame Scale; CoSS = Compass of Shame Scale; AO =attack others; AS = attack self; WD = withdrawal; AV = avoidance; K10 = Kessler Psychological Distress Scale.

Table S2.

Means, Standard Deviations (SD) and zero-order correlations between all measures used in the study (Men N = 421)

Variables (Men)	Mean (SD)	1	2	3	4	5	6	7	8	9	10	11
1. Age	33.72 (14.23)	-										
2. Education		−0.083	-									
3. Job Status		−0.184**	0.043	-								
4. ASQ ANX	45.63 (11.87)	−0.143**	0.037	0.027	-							
5. ASQ AVD	51.96 (10.68)	−0.071	−0.061	0.013	0.445**	-						
6. ESS	42.66 (13.27)	−0.292**	0.125*	0.054	0.601**	0.358**	-					
7. CoSS AO	10.35 (7.24)	−0.151**	0.081	−0.032	0.474**	0.365**	0.538**	-				
8. CoSS AS	16.68 (10.06)	−0.240**	0.115*	0.060	0.614**	0.350**	0.661**	0.558**	-			
9. CoSS WD	12.90 (9.26)	−0.182**	0.119*	0.028	0.617**	0.379**	0.658**	0.662**	0.728**	-		
10. CoSS AV	16.35 (7.77)	−0.210**	0.058	−0.016	0.340**	0.319**	0.427**	0.602**	0.526**	0.503**	-	
11. K10	20.32 (7.09)	−0.208**	0.133**	0.031	0.513**	0.368**	0.628**	0.406**	0.528**	0.520**	0.283**	-

Note. Education was coded 0 = high-school degree, 1 = bachelors' degree and above. Job status was coded 1 = employed, 0 = other. SD = Standard Deviation; ASQ = Attachment Style Questionnaire; ANX =- attachment anxiety; AVD = attachment avoidance; ESS = Experience of Shame Scale; CoSS = Compass of Shame Scale; AO = attack other; AS = attack self; WD = withdrawal; AV = avoidance; K10 = Kessler Psychological Distress Scale. * $p < 0.01$. ** $p < 0.001$

Table S3.

Means, Standard Deviations (SD) and zero-order correlations between all measures used in the study (Women N = 557)

Variables (Women)	Mean (SD)	1	2	3	4	5	6	7	8	9	10	11
1. Age	31.00 (12.78)	-										
2. Education		−0.275**	-									
3. Job Status		−0.123**	−0.019	-								
4. ASQ ANX	48.94 (12.23)	−0.223**	0.039	0.054	-							
5. ASQ AVD	50.70 (10.76)	0.033	−0.036	0.017	0.332**	-						
6. ESS	48.27 (14.10)	−0.290**	0.073	0.012	0.646**	0.269**	-					
7. CoSS AO	12.32 (7.30)	−0.101*	0.070	0.001	0.501**	0.290**	0.475**	-				
8. CoSS AS	21.01 (9.97)	−0.229**	0.096*	0.071	0.563**	0.186**	0.630**	0.489**	-			
9. CoSS WD	17.37 (9.29)	−0.159**	0.006	0.039	0.575**	0.298**	0.616**	0.628**	0.720**	-		
10. CoSS AV	17.11 (6.65)	−0.184**	0.000	0.087*	0.210**	0.256**	0.286**	0.427**	0.311**	0.379**	-	
11. K10	22.41 (7.57)	−0.101*	−0.014	0.059	0.570**	0.279**	0.633**	0.384**	0.536**	0.513**	0.156**	-

Note. Education was coded 0 = high-school degree, 1 = bachelors' degree and above. Job status was coded 1 = employed, 0 = other. SD = Standard Deviation; ASQ = Attachment Style Questionnaire; ANX =- attachment anxiety; AVD = attachment avoidance; ESS = Experience of Shame Scale; CoSS = Compass of Shame Scale; AO = attack other; AS = attack self; WD = withdrawal; AV = avoidance; K10 = Kessler Psychological Distress Scale. * $p < 0.01$. ** $p < 0.001$

Table S4

Fit Indices for Measurement Invariance Tests of the Hypothesized Model Among Men and Women

Model	χ^2	*df*	*p*	CFI	TLI	RMSEA (90%CI)	SRMR	ΔCFI
1. Unconstrained	40.322	12	0.0001	0.992	0.953	0.069 (0.047 0.094)	0.024	
2. Constrained	91.659	42	0.0000	0.986	0.976	0.049 (0.035 0.063)	0.053	0.006

Note. The following fit indexes are reported: χ^2=chi square; df=degrees of freedom; CFI=Comparative-fit-index; TLI=Tucker-Lewis-index; SRMR=Standardized-Root-Mean-Square-Residual; MC=model comparison; ΔCFI= CFI difference test.
